# Osteopontin regulates proliferation, apoptosis, and migration of murine claudin-low mammary tumor cells

**DOI:** 10.1186/s12885-016-2396-9

**Published:** 2016-06-10

**Authors:** S. Saleh, D. E. Thompson, J. McConkey, P. Murray, R. A. Moorehead

**Affiliations:** Department of Biomedical Science, Ontario Veterinary College, University of Guelph, 50 Stone Road East, Guelph, ON N1G2W1 Canada

**Keywords:** Osteopontin, Breast cancer, Claudin-low, Proliferation, Apoptosis, Migration

## Abstract

**Background:**

Osteopontin is a secreted phosphoglycoprotein that is expressed by a number of normal cells as well as a variety of tumor cells. With respect to breast cancer, osteopontin has been implicated in regulating tumor cell proliferation and migration/metastasis and may serve as a prognostic indicator. However it remains unclear whether osteopontin has the same impact in all breast cancer subtypes and in particular, osteopontin’s effects in claudin-low breast cancer are poorly understood.

**Methods:**

cDNA microarrays and qRT-PCR were used to evaluate osteopontin expression in mammary tumors from MTB-IGFIR transgenic mice and cell lines derived from these tumors. siRNA was then used to determine the impact of osteopontin knockdown on proliferation, apoptosis and migration *in vitro* in two murine claudin-low cell lines as well as identify the receptor mediating osteopontin’s physiologic effects.

**Results:**

Osteopontin was expressed at high levels in mammary tumors derived from MTB-IGFIR transgenic mice compared to normal mammary tissue. Evaluation of cell lines derived from different mammary tumors revealed that mammary tumor cells with claudin-low characteristic expressed high levels of osteopontin whereas mammary tumor cells with mixed luminal and basal-like features expressed lower levels of osteopontin. Reduction of osteopontin levels using siRNA significantly reduced proliferation and migration while increasing apoptosis in the claudin-low cell lines. Osteopontin’s effect appear to be mediated through a receptor containing ITGAV and not through CD44.

**Conclusions:**

Our data suggests that mammary tumors with a mixed luminal/basal-like phenotype express high levels of osteopontin however this osteopontin appears to be largely produced by non-tumor cells in the tumor microenvironment. In contrast tumor cells with claudin-low characteristics express high levels of osteopontin and a reduction of osteopontin in these cells impaired proliferation, survival and migration.

## Background

Osteopontin (OPN, *Spp1*) is a secreted glycophosphoprotein expressed by a number of cell types including endothelial cells, vascular smooth muscle cells, neural cells, epithelial cells, osteoblasts/osteoclasts, and immune cells [1–3]. OPN is involved in normal processes including wound healing, bone remodelling and inflammation as well as pathological processes such as cancer [4–7]. OPN mediates its actions through binding to integrins including αvβ1, αvβ3, αvβ5, αvβ6, α4β1, α5β1, α8β1, and α9β1 as well as CD44 [8–10].

In the mid-1990’s OPN mRNA and protein were found to be elevated in a number of different tumors compared to matching control tissue [11, 12]. Elevated levels of OPN have been found in tumors of the breast, prostate, colon, ovary, stomach, lung and liver [13–22]. OPN has been observed both in tumor cells themselves and in stromal cells surrounding the tumor [23]. More recent studies have shown that OPN is also elevated in the serum of breast cancer patients including those with early stage disease [24]. In breast cancer, OPN has been associated with poor prognosis [6, 7] and OPN has been shown to increase breast cancer cell survival and migration [25–27]. OPN is found in ER positive breast cancer and triple negative tumors [28].

Murine mammary tumor models have also been used to examine OPN’s role in breast tumorigenesis. A study investigating serum biomarkers in transgenic mice overexpressing an activated version of *c-neu* identified 3 proteins significantly elevated in tumor bearing mice compared to control mice and one of these proteins was OPN [29]. Interestingly, OPN was also able to discriminate tumor bearing mice from control mice when mammary tumor development was driven by a mutant p53 protein [29]. The tumors induced by the mutant p53 protein were estrogen receptor positive while the tumors induced by *c-neu* expression were estrogen receptor negative suggesting that OPN is elevated in mammary tumors with diverse characteristics [29].

In our mouse mammary tumor model, MTB-IGFIR transgenic mice develop mammary tumors due to elevated expression of the type I insulin-like growth factor receptor (IGF-IR) in mammary epithelial cells [30]. The mammary tumors that arise in this model have characteristics of human luminal breast cancer including expression of cytokeratin 8, cytokeratin 18 and E-cadherin however, these tumors cluster most closely with human basal-like breast cancer when gene expression profiles are used [31, 32]. Expression of the IGF-IR transgene in the MTB-IGFIR mice is controlled by a doxycycline inducible promoter and thus the impact of the loss of transgene expression in established mammary tumors can be evaluated. Loss of IGF-IR transgene expression in mammary tumors promotes regression followed by tumor re-growth in a subset of the mice. Mammary tumor recurrence in the absence of IGF-IR transgene expression is associated with epithelial to mesenchymal transition (EMT) [33] and tumors that cluster most closely with human claudin-low mammary tumors [31]. A number of cell lines have been generated from these tumors. RJ345 cells share characteristics with the luminal/basal like tumors while RJ348 and RM11A share characteristics with the claudin-low tumors [34, 35]

DNA microarray analysis comparing wild type mammary tissue to the mammary tumors revealed that *Spp1* was the most differentially expressed genes; *Spp1* was elevated 77-fold in the mammary tumors compared to normal mammary glands [31]. *Spp1* expression remained high in mammary tumors that acquired a more mesenchymal phenotype compared to normal mammary glands. Therefore, the purpose of this study was to further characterize the function of OPN in mammary tumorigenesis using murine mammary tumor cell lines and siRNA-mediated knockdown of OPN and its receptors.

## Methods

### Cell culture

The RM11A, RJ348 and RJ345 murine mammary tumour cells were grown in Dulbecco's modified eagle medium (DMEM) (Life Technologies Inc., Burlington, ON) containing the following supplements: 10 % tetracycline-free fetal bovine serum (FBS) (Clontech, Mountain View, CA), 1 mM sodium pyruvate, 10 mM 4-(2-hydroxyethyl)-1-piperazineethanesulfonic acid (HEPES), 4 mM glutamine, 2 mM hydrocortisone, 5 μg/ml estrogen, 5 μg/ml prolactin, 10 μg/ml EGF, 10 μg/ml insulin, 10 μg/ml doxycycline and 1 % antibiotic-antimycotic (Life Technologies Inc., Burlington, ON). Cells were maintained at 37 °C and 5 % carbon dioxide.

### RNA extraction

For tissue samples, flash-frozen tissues were homogenized using a handheld homogenizer in lysis/binding buffer from the *mir*Vana miRNA Isolation kit (Life Technologies Inc., Burlington, ON, Canada). For cell lines, cells were washed twice in ice cold PBS and the lysis/binding buffer from the *mir*Vana miRNA Isolation kit as added directly to the plate. The cells were scraped off using a cell scraper and the cell-buffer solution was then collected into 1.5 ml Eppendorf tubes. RNA from tissue and cell lines was extracted following the manufacturer’s protocol (enrichment for small RNAs was not performed). RNA was eluted with nuclease-free water and stored at -80 °C.

### Quantitative Real-Time PCR

RNA (500 ng) was reversed transcribed using iScript Reverse Transcription SuperMix (Bio-Rad Laboratories, Mississauga, ON, Canada) following the manufacturer’s protocol. The cDNA was then amplified using qScript cDNA SuperMix (Quanta Biosciences, Gaithersburg, MD) and the PrimePCR program on a CFX96 real-time PCR machine (Bio-Rad Laboratories, Mississauga, ON, Canada). All primers were purchased from Bio-Rad Laboratories (Mississauga, ON, Canada) and CFX Manager software v3.1 (Bio-Rad Laboratories, Mississauga, ON, Canada) was used to calculate expression levels and primer efficiencies. Primer efficiencies were as follows, *Spp1* – 104, *Itgav* – 101, *Itgb3* – 99, *Cd44* – 101, *Hprt* – 105, and *Ywhaz* – 110. The expression of *Spp1, Itgav, Itgb3* and *Cd44* were determined relative to the house-keeping genes *Hprt* and *Ywhaz* which were previously been shown to be suitable from a panel of 14 potential housekeeping genes [36].

### Immunohistochemisty

Tissue sections from formalin-fixed, paraffin-embedded mammary tumors were de-waxed with xylene and re-hydrated in 2 changes each of 100 %, 90 % and 70 % ethanol followed by incubation in PBS. Sections were blocked with 5 % BSA in Tris-buffered saline containing 0.1 % triton-X100 at room temperature for 30 min. The sections were then incubated with the OPN antibody (Ab8448, Abcam Inc, Toronto, ON) at a 1:200 dilution in PBS overnight at 4 °C. An anti-rabbit IgG (B7389, whole molecule) secondary was used at a 1:200 dilution in PBS for 1 h at room temperature (Sigma-Aldrich Canada Co, Oakville, ON). Sections were then stained with hematoxylin, dehydrated and mounted. Sections lacking the primary antibody were used as a control.

### Western blotting

Western blotting was performed as previously described [30]. Anti-OPN (AKm2A1; Santa Cruz Technologies, Santa Cruz, MA) and anti-β-actin (Cell Signaling Technology Beverly, MA) were used at a 1:1,000 dilution in 5 % skim milk in Tris-buffered Saline (TBS) containing 0.01 % Tween 20 (TBST). An anti-mouse secondary was used for detection of OPN while an anti-rabbit secondary was used for the detection of β-actin. Both secondary antibodies were obtained from Cell Signaling Technology (Beverly, MA) and were used at a 1:2,000 dilution in 5 % skim milk in TBST. Bands were visualized using Western Lightning Chemiluminescence substrate (Perkin Elmer, Wellesley, MA, USA) and a FluorChem 9900 gel documentation imaging system (Alpha Innotech, San Leandro, CA).

### Transient OPN Knockdown (siRNA)

Cells were transfected with stealth RNAi oligonucleotides directed against *Spp1*, *Cd44*, *Itgav* or a guanine-cytosine (GC) control sequence. All oligonucleotides were obtained from Life Technologies Inc. (Burlington, ON, Canada) and were used at a final concentration of 100 nM. Cells were transfected using Lipofectamine 2000 transfection reagent and Opti-MEM media (both obtained from Life Technologies Inc., Burlington, ON, Canada). After 4 h, fully supplemented media was added to the wells and cells were incubated at 37 °C with 5 % carbon dioxide.

### OPN Neutralizing Antibody

The mouse osteopontin neutralizing antibody (cat #AF808) was purchased from R&D Systems (Minneapolis, MN) and used at a concentration of 5 μg/μl or 10 μg/μl. A goat-anti-chicken IgG secondary antibody (Life Technologies, Burlington, ON, Canada) was used at 5 μg/ml or 10 μg/ml and served as a control.

### Immunofluorescence

Cells were plated onto sterile coverslips and were treated with either siRNA targeting OPN mRNA (described above) or an OPN neutralizing antibody (described above) for 48 h. Cells were then washed with PBS and fixed for 1 h at room temperature in 10 % buffered formalin. Coverslips with fixed cells were then washed with PBS and permeated with 0.1 % Triton X in PBS for 10 min at room temperature. Fixed cells were then washed once again with PBS, blocked in 5 % BSA for 10 min, and then incubated overnight at 4 °C with the primary antibody. Primary antibodies were used at a 1:200 dilution and anti-Ki67 (Abcam, Cambridge, MA) or anti-phospho-histone H3 (Abcam, Cambridge, MA) were used to identify proliferating cells while anti-cleaved caspase 3 (Millipore, Etobicoke, ON, Canada) was used to identify apoptotic cells. Fluorescent secondary antibodies were used a 1:100 dilution (Life Technologies, Burlington, ON, Canada) at room temperature for 2 h. Cells were then counterstained with 4',6-diamidino-2-phenylindole (DAPI) (Sigma, Oakville, ON, Canada) and mounted using Prolong Gold mounting media (Invitrogen, Burlington, ON, Canada). Images were captured with an Olympus BX-61 fluorescent microscope and positively stained cells were counted manually from at least 4 different, randomly selected fields of the coverslip.

### Cell counting

RJ348 cells were seeded in 6-well plates at a density of 200,000 cells per well. Four hours after seeding (RJ348 cells had attached to the plate at this point), RJ348 cells were treated with either siRNA targeting OPN mRNA (described above) or an OPN neutralizing antibody (described above) for 48 h. At this point cells were trypsinized, stained with typran blue and counted using a hemocytometer.

### Scratch wound migration assay

Cells where plated in 24-well culture dishes and grown to ~70-80 % confluency. Cells were then transfected with siRNA targeting *Spp1*, *Cd44*, or *Itgav*, or a guanine-cytosine control sequence as described above. Twenty-four hours post siRNA transfection a scratch was induced using a pipette tip and images were captured immediately after scratch induction as well as 24 h or 48 h after scratch induction. Images were captured using an Olympus IX71 inverted microscope (Toronto, ON, Canada) and Q-capture software (Surrey, BC, Canada). Image J software [37] was used to quantify the percent wound closure.

### Boyden chamber assay

Matrigel (Life Technologies, Burlington, ON, Canada) was diluted 1:6 in DMEM (Life Technologies, Burlington, ON, Canada) and 20 μl of diluted matrigel was plated on to the upper compartment of a Falcon cell culture insert (cat #353097; BD Bioscience, Mississauga, ON, Canada). Approximately 1x10^5^ cells were cultured in 200 ul of serum-free media in the upper compartment of the insert. The bottom well was filled with 300 ul of media containing serum. Cells were cultured at 37 °C and 5 % carbon dioxide for 20 h. Media was then aspirated from the lower chamber and the bottom of the insert was fixed with 5 % glutaraldehyde in 1xPBS, for 10 min, washed with water and stained with 0.5 % toluidine blue staining solution 10-20 min at room temperature. The inner surface of the upper chamber was then wiped clean and cells that had migrated to the bottom of the insert were visualized using an Olympus IX71 inverted microscope (Toronto, ON, Canada) and Q-capture software (Surrey, BC, Canada). The number of cells on the bottom of the insert were counted manually.

### Statistics

A paired student’s t test was used to compare means from the treated and control groups. Differences were considered to be significant at *p* < 0.05.

## Results

### OPN expression in mammary tissue, mammary tumors and mammary tumor cell lines

A previous study from our lab described the DNA microarray analysis of wild-type mammary tissue (WT), primary mammary tumors (PMT) and recurrent spindle tumors (RST) generated in MTB-IGFIR transgenic mice [31]. PMTs were induced by the transgenic expression of the type I insulin-like growth factor receptor (IGF-IR) in mammary epithelial cells of MTB-IGFIR transgenic mice while RSTs resulted following the downregulation of the IGF-IR transgene in PMTs. As described in Franks et al. [31], the most differentially expressed gene between wild type mammary tissue and primary mammary tumors was *Spp1* or OPN and this was confirmed by qRT-PCR. Raw and normalized gene expression data is available in the Gene Expression Omnibus (GEO:GSE32152).

**Fig. 1:**
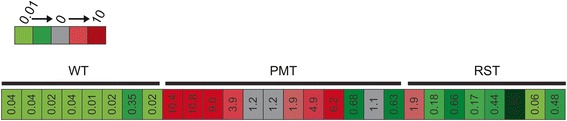
Heat map illustrating the expression of OPN in 8 wild type mammary glands (WT), 12 primary mammary tumors (PMT) and 8 recurrent spindle tumors (RST) that arose in MTB-IGFIR transgenic mice as determined by cDNA microarray analysis. Numerical values in the box represent OPN expression relative to a universal mouse reference RNA sample. Microarrays were performed using Agilent Whole Mouse Genome 4 × 44 k Gene Expression Arrays (product number G4122F, Agilent) as described in [31]

Figure 1 shows the range of *Spp1* expression in 8 WT, 12 PMT and 8 RST samples as determined by the DNA microarray. Each of these tissues/tumors were obtained from different mice. The WT samples consistently expressed only very low levels of *Spp1* while *Spp1* expression in the tumor samples was more variable. Table 1 shows the average *Spp1* expression as determined by the cDNA microarray. Quantitative RT-PCR was also performed on 4 WT, 4 PMT and 4 RST samples. As shown in Table 1, although the values were higher for each group, qRT-PCR confirmed the cDNA microarray data in that the PMTs had the highest level of *Spp1* with the RSTs having less *Spp1* than the PMTs but more than the WT samples which only expressed very low levels of *Spp1*.

**Table 1:** OPN expression in tumors and tissue

Sample	*Spp1* (DNA Microarray)^a^	*Spp1* (qRT-PCR)^b^
WT	0.07 ± 0.04	1.3 ± 0.92
PMT	4.33 ± 1.13	675.2 ± 212.4
RST	0.59 ± 0.02	94.7 ± 48.7

^a^OPN expression relative to a murine reference DNA sample, WT (*n* = 8), PMT (*n* = 12), RST (*n* = 8)

^b^OPN expression relative to housekeeping genes *Hprt* and *Ywhaz*, WT (*n* = 4), PMT (*n* = 4), RST (*n* = 4)

**Fig. 2:**
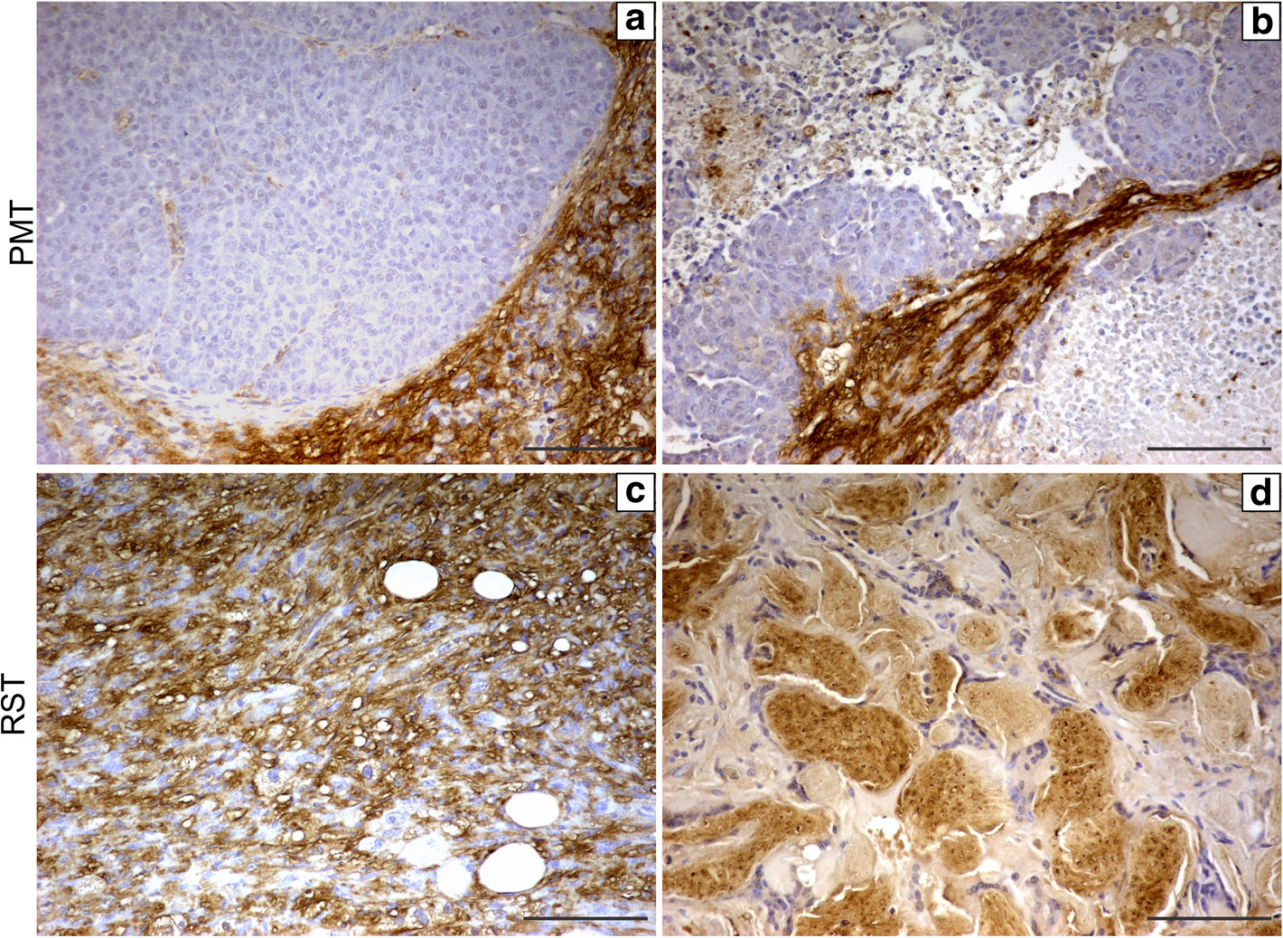
Immunohistochemistry for OPN protein in (**a**, **b**) two independent PMT tumors and (**c**, **d**) two independent RST tumors. The brown staining indicates OPN protein which was expressed at only low levels in tumors cells of PMTs but at higher levels in the tumors cells or RSTs. RST tumors varied in histology with some tumors being composed primarily of spindle-shaped cells (**c**) and other tumors composed primarily of cells with squamous characteristics (**d**). Scale bars 100 μM

OPN was also evaluated in PMTs and RSTs using immunohistochemistry. As shown in Fig. 2, OPN protein in PMTs was primarily expressed by stromal cells surrounding the tumor cells with only low, sporadic staining of OPN protein being observed in the tumor cells themselves (Fig. 2a, b). In contrast, high levels of OPN protein were frequently found in the tumor cells of RSTs (Fig. 2c, d). Sections lacking the primary antibody did not display any staining (data not shown).

### OPN expression in murine mammary tumor cell lines

To determine whether the tumor cells themselves were producing OPN, cell lines derived from different MTB-IGFIR mammary tumors were evaluated. The two best characterized lines are RJ345 cells (which have characteristics similar to PMT tumors in MTB-IGFIR transgenic mice) and RJ348 cells (which have characteristics similar to RST tumors in MTB-IGFIR transgenic mice) [34, 38–40]. As shown in Table 2, RJ348 cells expressed higher levels of *Spp1* than RJ345 cells. To confirm that murine mammary tumor cells with characteristics of human claudin-low breast cancer [34] express high levels of *Spp1*, another cell line, RM11A, which was derived from a different RST tumor was also examined. As shown in Table 2, RM11A cells expressed higher levels of *Spp1* than the RJ345 cells. All the tumor cell lines expressed higher levels of *Spp1* than the wild-type mammary tissue but lower levels than the PMTs (Table 1). Since RM11A cells expressed the highest level of *Spp1* we collected conditioned media from these cells and confirmed through western blotting that OPN was detectable in conditioned media of RM11A cells (data not shown).

**Table 2:** OPN expression in mammary tumor cell lines

Sample	*Spp1* (qRT-PCR)^a^
RJ348	21.8 ± 5.7
RJ345	6.4 ± 1.9
RM11A	89.5 ± 13.2

^a^ *Spp1* expression relative to housekeeping genes *Hprt* and *Ywhaz*, RJ348 (*n* = 4), RJ345 (*n* = 4), RM11A (*n* = 4)

### *Spp1* knockdown decreases proliferation and increases apoptosis

**Fig. 3:**
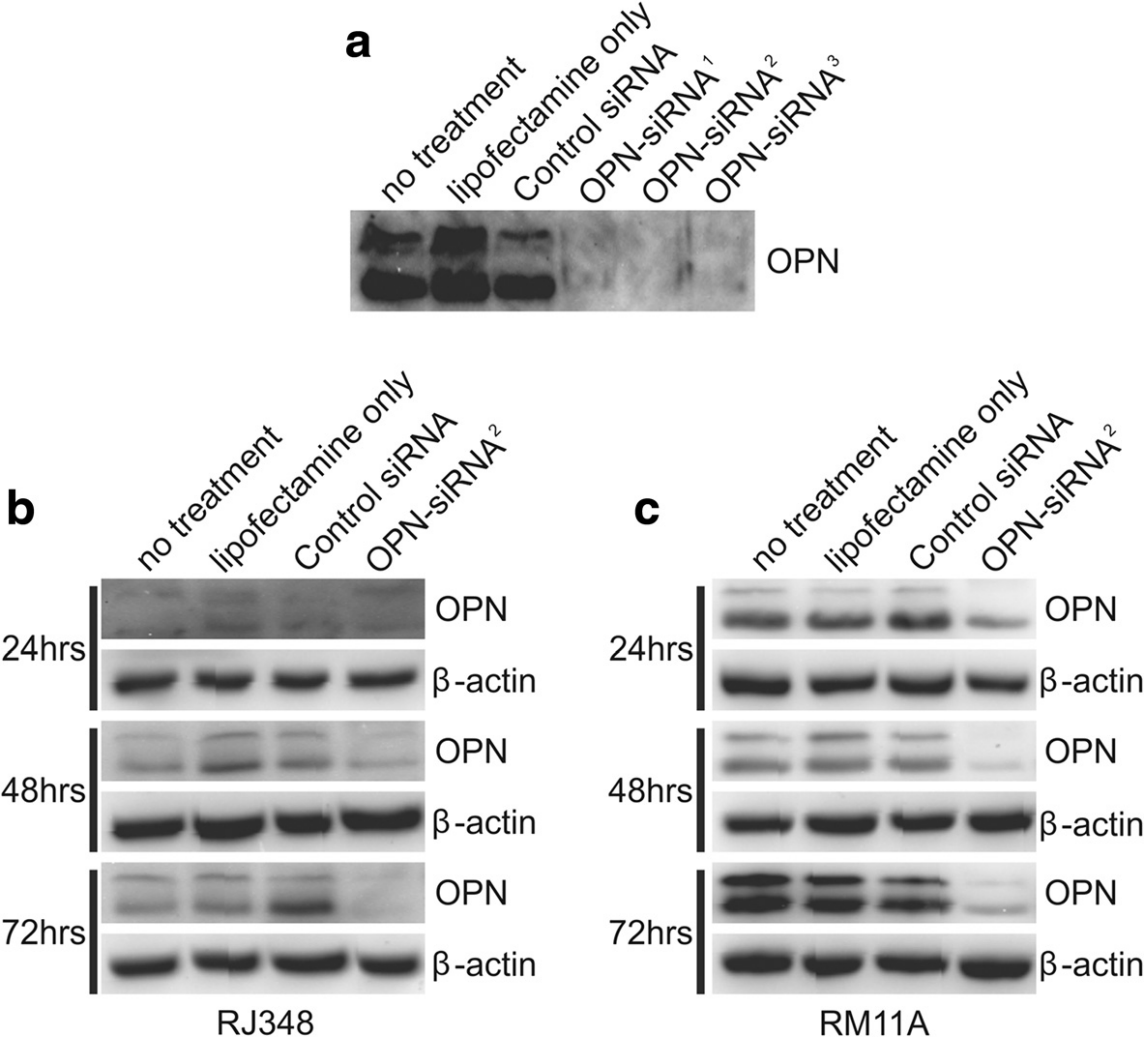
Representative western blots showing OPN protein levels in (**a**) RM11A cell following no treatment, treatment with lipofectamine only, treatment with a GC control siRNA or 3 different siRNAs targeting different regions on OPN mRNA. Representative western blots of OPN protein level following no treatment, treatment with lipofectamine only, treatment with a GC control siRNA or treatment with OPN- siRNA^2^ in (**b**) RJ348 cells and (**c**) RM11A cells 24, 48 or 72 h after transfection

Since the claudin-low mammary tumor cell lines expressed higher levels of *Spp1* and little is known about the function of OPN in claudin-low breast cancer, OPN function was evaluated in the claudin-low cell line RJ348. OPN was detected as two bands with molecular weights of ~62 kDa and ~58 kDa and three different siRNA oligonucleotides targeting different regions of OPN mRNA suppressed both the 62 kDa and 58 kDa OPN bands (Fig. 3a). A time course was then performed using OPN-siRNA^2^ in RJ348 cells and it was observed that OPN knockdown was achieved 48 h after RNAi treatment and OPN protein levels remained low 72 h after OPN RNAi treatment (Fig. 3b). OPN protein levels were reduced approximately 60 % following OPN siRNA treatment. The efficacy of OPN-siRNA^2^ was confirmed in RM11A cells (Fig. 3c).

**Fig. 4:**
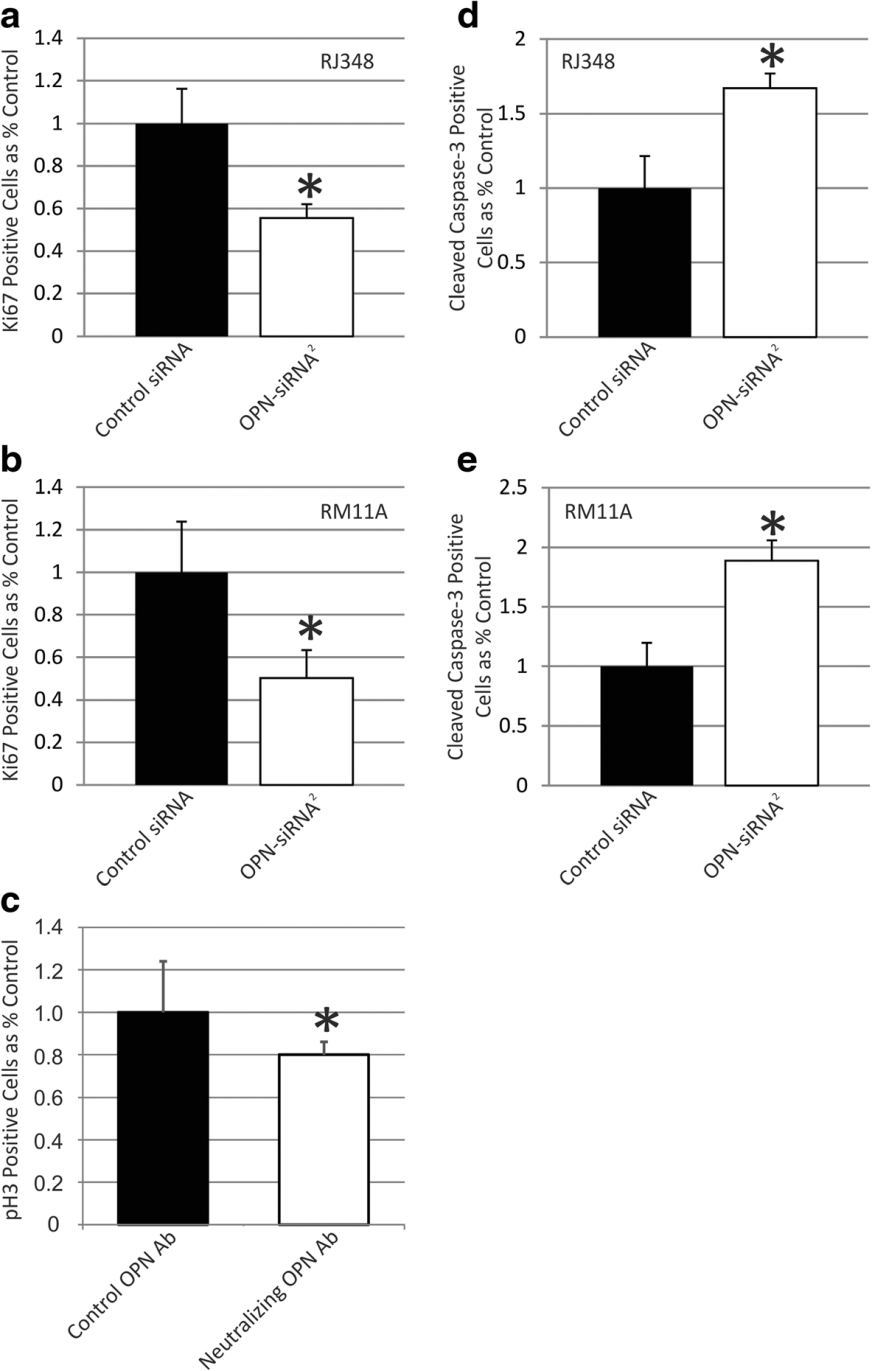
Quantification of (**a**, **b**) Ki67 immunofluorescence or (**d**, **e**) cleaved caspase 3 immunofluorescence in (**a**, **d**) RJ348 cells or (B,E) RM11A cells after treatment with a GC control siRNA or OPN-siRNA^2^. The percentage of Ki67 positive or cleaved caspase 3 positive cells following OPN-siRNA^2^ treatment are presented relative to cells treated with the GC control siRNA. **c** phospho-histone H3 immunofluorescence in RJ348 cells following administration of 5 μg of an OPN neutralizing antibody or 5 μg of a control antibody. The percentage of phospho-histone H3 positive cells following OPN neutralizing antibody treatment is presented relative to the control antibody. A paired t-test was used to determine statistical significance, *p˂0.05, n ≥ 3

Proliferation was evaluated 48 h after OPN-siRNA^2^ transfection using immunofluorescence for Ki67. OPN knockdown produced a significant reduction in Ki67 positive staining in both RJ348 (Fig. 4a) and RM11A (Fig. 4b) cells compared to cells treated with the control siRNA. Proliferation was also evaluated using immunofluorescence for phosphorylated histone H3 and manual cell counting using trypan blue exclusion. Both of these experiments also showed a significant decrease in proliferation in RJ348 cells treated with OPN-siRNA^**2**^ compared to cells treated with the control siRNA (data not shown). As an alternative approach, RJ348 proliferation was assessed following incubation with 5 μg of an OPN neutralizing antibody. As shown in Fig. 4c, treatment with an OPN neutralizing antibody also significantly reduced RJ348 proliferation, albeit to a lesser extent. Treating the RJ348 cells with 10 μg of OPN neutralizing antibody resulted in a similar 20 % reduction in cell proliferation (data not shown).

Apoptosis was evaluated 48 h after OPN-siRNA^**2**^ transfection using immunofluorescence for cleaved caspase 3. OPN knockdown produced a significant increase in cleaved caspase 3 positive cells in both RJ348 (Fig. 4d) and RM11A (Fig. 4e) cells compared to cells treated with the control siRNA.

### Migration and invasion were reduced following OPN knockdown

**Fig. 5:**
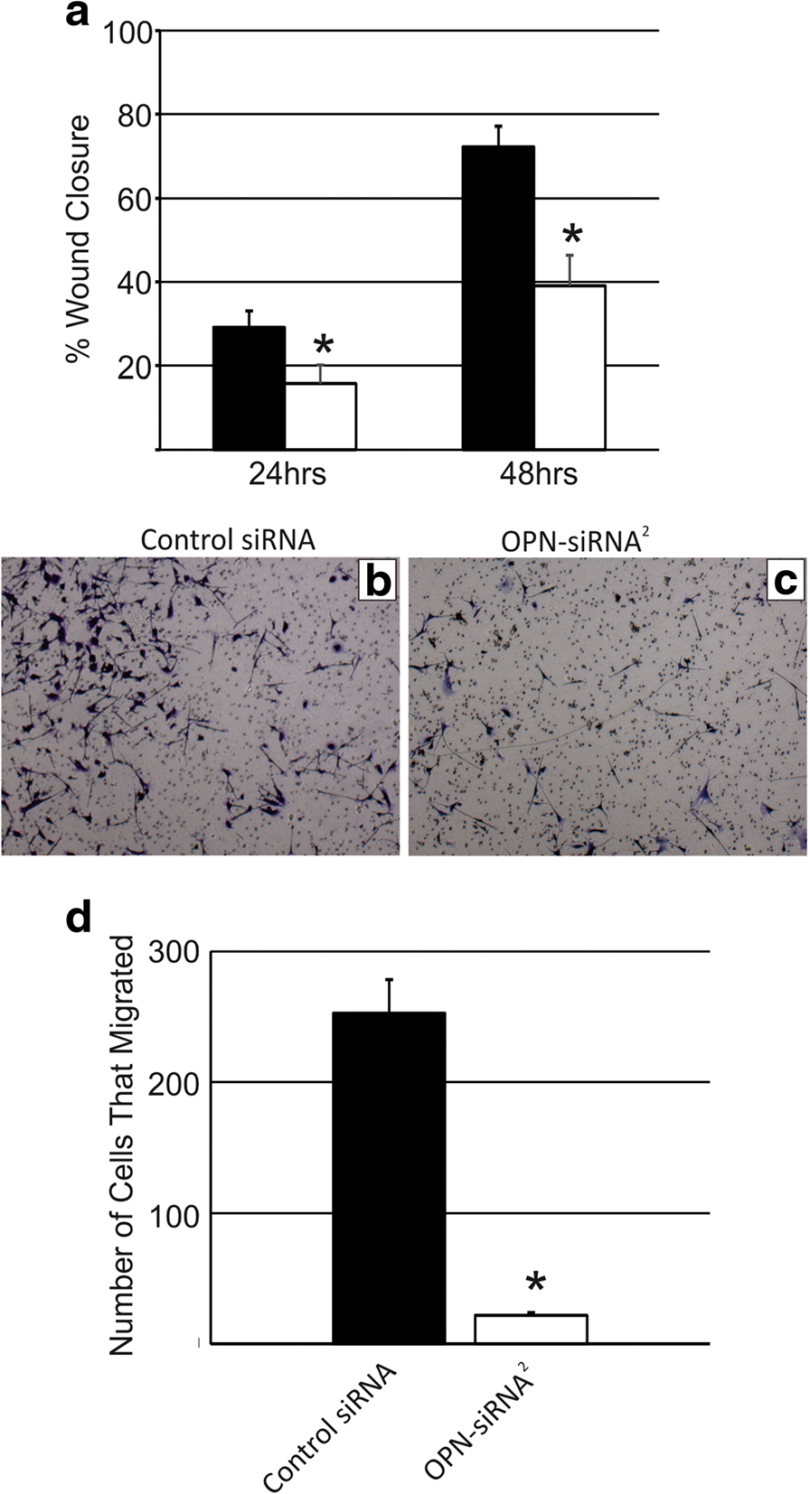
Quantification of (**a**) RJ348 cell migration following treatment with a GC control siRNA or OPN-siRNA^2^ as determined by a scratch wound assay 24 or 48 h after scratch induction. Representative images of RJ348 cells that migrated to the opposite side of a transwell assay following treatment with (**b**) GC control siRNA or (**c**) OPN-siRNA^2^. The bars represent mean ± SEM. A paired T-test was used to determine statistical significance, *p˂0.05, *n* = 3. (**d**) Quantification of the number of cells that migrated in the transwell assay after treatment with a GC control siRNA or OPN-siRNA^2^. The bars represent mean ± SEM. A paired t-test was used to determine statistical significance, *p˂0.05, *n* ≥ 3

Cell migration was initially assessed using a scratch wound assay. RJ348 cells treated with OPN-siRNA^**2**^ had significantly impaired migration compared to RJ348 cells treated with the control siRNA at both the 24 and 48 h time points (Fig. 5a). Cell migration was also assessed using a transwell assay. Similar to the scratch wound assay, OPN knockdown significantly reduced RJ348 cell migration in the transwell assay (Fig. 5b-d). Scratch wound assays are difficult to perform using the RM11A cells as these cells have a disperse growth pattern and do not tightly pack together. Therefore migration and invasion was only evaluated in the RJ348 cells.

### Proliferation and migration following OPN receptor knockdown

The two main receptors reported for OPN are CD44 and integrin α_V_β_3_, however, other integrin receptors are also involved. Quantitative RT-PCR of *Cd44*, integrin α_V_ (*Itgav*), and integrin β_3_*(Itgb3*) mRNA expression revealed that only *Cd44* and *Itgav* where highly expressed in RJ348 cells (Table 3). Similar levels of expression were observed in RM11A cells (Table 3).

**Table 3:** *Itgav*, *Itgb3* and *Cd44* expression in mammary tumor cell lines

	*Itgav* ^a^	*Itgb3* ^a^	*Cd44* ^a^
RJ348	0.92 ± 0.14	0.012 ± 0.004	0.99 ± 0.12
RM11A	0.91 ± 0.11	0.013 ± 0.003	0.91 ± 0.11

^a^OPN expression relative to housekeeping genes, *Hprt* and *Ywhaz*

To determine whether CD44 or integrin α_V_ were mediating the effects of OPN in RJ348 cells, these proteins were transiently knocked down using siRNA (RM11A cells were not evaluated). Quantitative RT-PCR revealed that *Cd44* expression could be reduced to 12 % of the control RNAi treated RJ348 cells (88 % knockdown) while *Itgav* expression could be reduced to 32 % of control RNAi treated RJ348 cells (68 % knockdown).

**Fig. 6:**
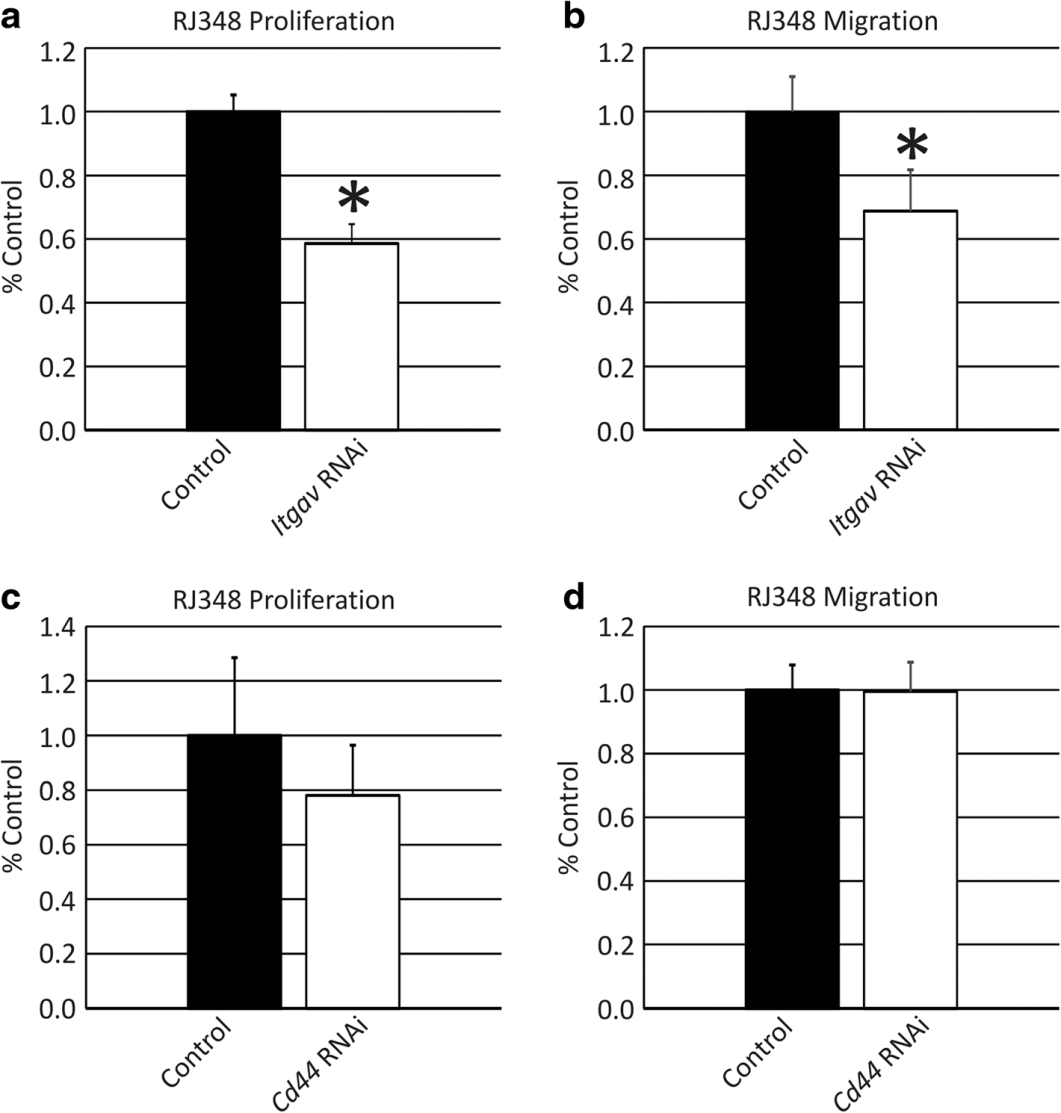
Quantification of (**a**, **c**) phospho-histone H3 immunofluorescence, (**b**) transwell migration and (**d**) scratch wound closure in RJ348 cells following treatment with (**a**, **b**) siRNA targeting *Itgav* or (**c**, **d**) siRNA targeting *Cd44*. Both proliferation and migration are presented relative to a GC control siRNA. *p˂0.05, *n* ≥ 3

*Itgav* knockdown significantly reduced proliferation in RJ348 cells (Fig. 6a) while *Cd44* knockdown induced a small, non-significant reduction in RJ348 proliferation (Fig. 6c). With respect to migration, *Cd44* knockdown did not significantly reduce wound closure (Fig. 6d). *Itgav* knockdown appeared to negatively impact cell adhesion in the scratch wound assay and thus only a transwell assay was performed. *Itgav* knockdown significantly reduce RJ348 transwell migration (Fig. 5b).

## Discussion

Our lab has generated a transgenic model of mammary tumorigenesis. In this model, human IGF-IR is expressed in mammary epithelial cells in a doxycycline inducible manner and IGF-IR transgene expression induces mammary tumor development [30]. Down-regulation of IGF-IR transgene (through doxycycline withdrawal) in established mammary tumors results in the regression of most of the mammary tumors and tumor recurrence in a subset of mice. Some of the recurrent mammary tumors acquire a spindle-like morphology and no longer express the IGF-IR transgene [33]. Gene expression analysis and clustering with human breast cancers revealed that the IGF-IR induced mammary tumors (also known as PMTs) express markers of luminal tumors but cluster closely with human basal-like tumors with the recurrent mammary tumors (also known as RSTs) express markers of claudin-low tumors and cluster closely with human claudin low breast cancers [31]. Claudin-low mammary tumors are a subset of basal-like breast cancers that are typically estrogen receptor, progesterone receptor and HER2 negative, express low levels of claudins 2, 4 and 7 and have characteristics of progenitor cells [41–43]. Since the most differentially expressed gene between normal mouse mammary tissue and IGF-IR induced mammary tumors identified in our previous study was *Spp1*, this gene was further examined in this current manuscript in the transgenic model and cell lines derived from the IGF-IR transgenic mice (MTB-IGFIR transgenic mice).

Using cell lines derived from a PMT or two different RSTs from the MTB-IGFIR transgenic mice we found that the RST-derived cell lines (RJ348 and RM11A) had higher expression of OPN than the PMT-derived cell line (RJ345). This finding was somewhat surprising considering that PMTs in MTB-IGFIR transgenic mice had higher OPN expression than RSTs and may suggest that most of the OPN in the PMTs is produced by non-tumor cells in the tumor microenvironment while the tumor cells themselves are the main source of OPN in the RSTs.

Immunohistochemical staining for OPN supported this theory as positive OPN staining in tumor cells from PMTs was typically low and sporadic, however, intense OPN staining was observed in stromal cells surrounding PMTs. In contrast, tumor cells in RSTs more frequently stained positive for OPN protein than tumor cells of PMTs. Studies in human breast cancer support this finding in that OPN was negatively correlated with luminal breast cancer subtypes [44] (no studies evaluating OPN expression in human claudin-low tumors have been described). Moreover, the human breast cancer cell line, MCF-7, which possesses characteristics of luminal breast cancer express lower levels of OPN than the human claudin-low breast cancer cell line, MDA-MB-231 [45]. Therefore, luminal tumors may depend on OPN from the microenvironment while claudin-low tumors may produce OPN.

We focused on the murine claudin-low mammary tumor cell lines since (1) the claudin-low murine mammary tumor cells expressed high levels of OPN, (2) claudin-low tumors are poorly understood, and (3) claudin-low tumor do not respond well to conventional therapies and thus alternative therapeutic strategies for this type of tumor requires identification. Our findings demonstrate that claudin-low mammary tumor cells rely on OPN for proliferation, survival and migration as knockdown of OPN using siRNA inhibited proliferation and migration while increasing apoptosis. An OPN neutralizing antibody was also capable of significantly inhibiting RJ348 proliferation albeit, less efficiently than OPN knockdown (apoptosis was not evaluated). These findings are consistent with studies on MDA-MB-231 cells which showed that disruption of OPN function impaired proliferation [46, 47], survival [47–49] and migration [46, 48].

In an attempt to determine which receptors OPN was interacting with, the two best-characterized OPN receptors, CD44 and integrin αvβ3 (ITGAV and ITGB3) [50] were examined. While RJ348 cells expressed considerable amounts of *Cd44* and *Itgav* mRNA, only very low levels of *Itgb3* were detected. To evaluate receptor function, *Cd44* or *Itgav* mRNA was knocked down using siRNA. Only knockdown of *Itgav* and not *Cd44* significantly decreased cell proliferation and migration suggesting that *Itgav* in association with a β-integrin, other than β3, is mediating OPN’s effects in RJ348 cells. OPN can also bind to αv containing integrins αvβ1,αvβ5, and αvβ6 [8–10] and thus in RJ348 cells, OPN is likely mediating at least some of its effects via one of these integrin receptors. RNA sequencing has been performed on the RJ348 cells and *Itgb1* and *Itgb5*, but not *Itgb6* are highly expressed in RJ348 cells and thus presumably one of these two β-integrins are interacting with *Itgav* to mediate OPN signaling. The only published study that directly manipulated OPN receptors in human breast cancer utilized a CD44 neutralizing antibody in MDA-MB-231 and they observed that antibody mediated suppression of CD44 signaling inhibited migration [46]. Therefore, it remains unclear which receptors mediate the physiologic effects of OPN in claudin-low breast cancer.

## Conclusions

Despite numerous studies implicating OPN in a variety of cancers, therapeutic strategies targeting OPN function have not materialized (OPN is being evaluated as a biomarker for a number of human cancers including non-small cell lung cancer, head and neck cancer, and pancreatic cancer (www.clinicaltrials.gov)). Our work, combined with published studies on MDA-MB-231 cells, argue that targeting OPN in claudin-low breast cancers may prove to be an effective therapeutic approach however a more complete understanding of the OPN-receptor interactions as well as development of potent/specific OPN inhibitors are required to translate this strategy from the pre-clinical to clinical setting.

## Abbreviations

*Cd44*, gene name for Cd44; CD44, protein name for Cd44; EGF, epidermal growth factor; GC, guanine-cytosine; IGF-IR, type I insulin-like growth factor receptor; *Itgav*, gene name for integrin α_v_; ITGAV, protein name for integrin α_v_; *Itgb3*, gene name for integrin β3; MTB-IGFIR, transgenic mouse expressing human IGF-IR in mammary epithelial cells in response to doxycycline; OPN, osteopontin protein; PMT, primary mammary tumor from MTB-IGFIR transgenic mouse; qRT-PCR, quantitative reverse transcription polymerase chain reaction; RJ345, murine mammary tumor cell line with luminal characteristics; RJ348, murine mammary tumor cell line with claudin-low characteristics; RM11A, murine mammary tumor cell line with claudin-low characteristics; RST, recurrent spindle tumor from MTB-IGFIR transgenic mouse; siRNA, small interfering ribonucleic acid; *Spp1*, gene name for osteopontin
